# Effects of Rutile–TiO_2_ Nanoparticles on Accelerated Weathering Degradation of Poly(Lactic Acid)

**DOI:** 10.3390/polym12051096

**Published:** 2020-05-11

**Authors:** Ana Antunes, Anton Popelka, Omar Aljarod, Mohammad K. Hassan, Adriaan S. Luyt

**Affiliations:** Center for Advanced Materials, Qatar University, P.O. Box 2713 Doha, Qatar; ana.antunes@qu.edu.qa (A.A.); anton.popelka@qu.edu.qa (A.P.); o.y.aljarod@qu.edu.qa (O.A.); mohamed.hassan@qu.edu.qa (M.K.H.)

**Keywords:** poly(lactic acid), rutile titanium (IV) dioxide, accelerated weathering degradation, morphology and properties

## Abstract

The effect of accelerated weathering on poly(lactic acid) (PLA) and a PLA nanocomposite with rutile titanium (IV) dioxide (rutile–TiO_2_) was investigated. The accelerated weathering test applied consecutive steps of ultraviolet (UV) (at 340 nm and 0.76 W m^−2^ irradiance) and moisture at 50 °C for 2000 h, following the ASTM D4329 standard. The morphology, chemical structure, molecular weight, crystallization, as well as mechanical and thermal properties were thoroughly studied. Samples were characterized after 500 h, 1000 h and 2000 h exposure. Different degradation mechanisms were proposed to happen during the weathering exposure and confirmed based on the experimental data. The PLA and PLA/TiO_2_ surfaces presented holes and increasing roughness over the exposure time. The molecular weight of the weathered samples decreased due to chain scission during the degradation processes. Thermal stability decreased in the presence of TiO_2_ and a double melting peak was observed for the PLA/TiO_2_ nanocomposite. A general improvement in the mechanical properties of the PLA/TiO_2_ nanocomposite was observed over time during the accelerated weathering analysis up to 1000 h of exposure time. After 2000 h of weathering exposure, the PLA and PLA/TiO_2_ became extremely brittle and lost their ductile properties. This was ascribed to a significant increase in the degree of crystallinity upon weathering, which was accelerated in the presence of TiO_2_. Atomic force microscopy (AFM) using amplitude modulation–frequency modulation (AM–FM) tool confirmed the mechanical changes in the surface area of the PLA samples after accelerated weathering exposure. The stiffness and Young’s modulus achieved higher values than the unweathered ones up to 1000 h of exposure time. The changes in the physical and chemical properties of PLA/TiO_2_ over the ageing time confirm the photocatalytic activity of rutile–TiO_2_.

## 1. Introduction

Poly(lactic acid) (PLA) is an aliphatic thermoplastic polyester considered to be environmentally friendly. It is derived from renewable plant resources, presents properties comparable to petrochemical-based polymers, and it is biodegradable [1,2,3,4]. This polymer shows good physico-mechanical properties, low thermal stability and poor hydrophilicity [5]. PLA is useful for different application areas such as medical uses, pharmaceutical-controlled release and biodegradable packaging because of its excellent biocompatibility and biodegradability under appropriate conditions [6,7,8]. However, this polymer has a very slow biodegradation rate at temperatures less than its glass transition [2,8,9,10]. A period longer than 2 months is required for the decomposition of PLA in underground conditions (humidity and microorganisms) [11]. Some studies were done to thoroughly investigate the degradation process of PLA and how it can be accelerated or delayed according to final uses. Photo [12,13,14,15], hydrolytic [14,15,16,17,18,19] and thermal [2,9,15,20,21] degradation have been explored, as well as soil and enzymatic degradation [2,21,22,23]. Some previous studies show that the degradation rate can be controlled by loading additives such as ZnO, MgO and CaO [24,25,26]. The purpose of using nanoparticles is to modify various properties such as hydrolytic, mechanical and thermal stability, which control the degradation process in different media [2,22,27,28].

Titanium (IV) dioxide (TiO_2_) is nowadays considered to be one of the most interesting nanomaterials. It is relatively cheap, nontoxic, readily available, and it presents antibacteriostatic and photocatalytic activities. A fair amount of research report that by using TiO_2_ nanoparticles in organic polymer nanocomposites, a significant rise in degradability under solar or UV irradiation could be accomplished. The degradation rates of a PLA matrix, in particular, can be significantly improved [2,5].

TiO_2_ exists in three morphologic crystalline forms: anatase, rutile and brookite. The first form is widely used for photo-catalytic and degradation studies [29,30,31]. Zhuang et al. [5] studied PLA/TiO_2_ nanocomposites with different contents of TiO_2_. Different property changes were observed, such as improvements in the thermal and mechanical properties at 3 wt % of TiO_2_ and more extensive degradation when exposed to UV light. A degradation analysis of PLA and PLA/TiO_2_ composites also showed that the hydrolytic degradation of PLA can initially be accelerated in the presence of TiO_2_, but that the crystallinity increased with increasing degradation, which again slowed down the degradation process [18]. Wang et al. [32] reported that both the rutile and anatase TiO_2_ affected the UV degradation of PLA. However, the anatase–TiO_2_ particles caused photo-degradation of PLA faster than the rutile–TiO_2_ particles. Man et al. [33] also compared the degradation process of PLA/anatase–TiO_2_ and PLA/rutile–TiO_2_. The UV absorbance results showed a UV shielding effect of rutile–TiO_2_ and a degradation acceleration effect of anatase–TiO_2_ when the particles were surrounded in the PLA matrix. Only a few studies were published using rutile–TiO_2_. The behaviour of rutile–TiO_2_ after a long period of degradation exposure is also not known.

Some studies were done on PLA loaded with different additives to investigate the weathering degradation using accelerated tests [34,35,36,37,38]. Accelerated weathering tests help to predict the materials’ degradation under specific conditions in a shorter period of time. Two different accelerated ageing methods of PLA were compared by Litauszki et al. [37]. The goal was to create a possible correlation between two methods: the accelerated weathering chamber and the photodegradation with a homogenized laser beam. Chávez-Montes et al. [39] did it between natural and artificial weathering and showed that it is possible to correspond 10 to 68 days of natural weathering to shorter artificial exposure times of 56 h and 360 h, respectively. They tested amorphous and semicrystalline PLA nanocomposites with 5 wt % of montmorillonite, and exposed it to the artificial weathering degradation for periods of up to 360 h. It was observed that the resistance to artificial weathering degradation was more pronounced in the semicrystalline than in the amorphous PLA and that the addition of clay favoured the degradation of PLA, decreasing the nanocomposites’ molecular weight after weathering. Kaynak and Sari [34] compared the effect of accelerating weathering on neat PLA and PLA with 1 wt % of montmorillonite. A significant decrease in the molecular weight and reductions in the mechanical properties of the specimens were observed for PLA. Spiridon et al. [35] studied the degradation under accelerated weathering for 600 h of PLA with embedded grape seeds. The authors found a stabilizing effect against UV exposure after exposure of the materials to the combined action of temperature, humidity and UV radiation. Varsavas and Kaynak [40] explored the degree of degradation of the PLA structure in accelerated weathering conditions of both UV-irradiation and moisture cycles for periods of 400 h when it is reinforced with 15 wt % glass fibers. Their results revealed alterations in the structure and properties of the specimens due to a significant decrease in the molecular weight of the PLA, which was much more critical in neat PLA than in the composite. Inorganic glass fibers in PLA showed no significant degradation during weathering, and their reinforcement of the polymer did not change. Kaynak and Kaygusuz [41] explored the consequences of accelerated weathering on the behaviour of PLA/halloysite composites after exposure to humidity and UV irradiation for a total duration of 300 h. Their results revealed significant and random chain scission of PLA during photolysis and partial hydrolysis degradation, decreasing the mechanical properties. Cong et al. [42] showed that the presence of TiO_2_ nanoparticles promoted the degradation of poly(ethylene-co-vinylacetate) EVA/PLA/TiO_2_ blends under accelerated weathering for cyclic periods of a total of 672 h. After the testing, the tensile properties and thermal stability of the blends showed a considerable reduction, and many micro-holes were observed on the surface of the collected samples.

Many researchers reported the use of TiO_2_ nanoparticles in polymeric matrices, but the investigations on PLA/TiO_2_ nanocomposites mainly focused on UV degradation through exposure to natural sunlight. Only a few papers explored the use of an accelerated weathering machine to study the degradation of PLA over long periods. TiO_2_ (particularly the rutile type) and artificial weathering need a deeper understanding to enhance the biodegradability of PLA compared to natural sunlight exposed weathering. The aim of the work reported in this study is to examine the effect of rutile–TiO_2_ on the physio-chemical properties of a PLA/TiO_2_ nanocomposite and to evaluate its influence on the degradation process under accelerated weathering for long periods of time. The weathering of PLA and the PLA/TiO_2_ nanocomposite was carried out with alternating cycles of UV light and moisture at controlled temperatures for cycles of 500 h up to 2000 h. The samples were characterized to assess the extent of thermal, hydrolytic and UV-degradation of the PLA nanocomposite loaded with 3 wt % of rutile–TiO_2_.

## 2. Experimental

### 2.1. Materials

The PLA used in this study is a high molar mass biopolymer (Ingeo™ Biopolymer 2003D), obtained from NatureWorks, LLC (Minnetonka, MN, USA). It is transparent with a density of 1.24 g cm^−3^, melt flow index of 6.0 g/10 min (2.16 kg and 210 °C), glass transition of ~55 °C and a melting temperature of ~150 °C.

The rutile–TiO_2_ nanopowder, with particle sizes < 100 nm and 99.5% purity, was obtained from Wuhan Pensieve Technology Co., Ltd. (Wuhan, China).

### 2.2. Preparation Method

The polymer was first dried in an oven at 60 °C overnight prior to sample preparation. The nanocomposite with 3 wt % of TiO_2_ was prepared by melt-mixing the rutile–TiO_2_ nanopowder with the polymer using a Plastograph EC (Brabender GmbH, Duisburg, Germany) at 170 °C and 30 rpm screw speed for ten minutes. The samples were then compression molded into 1 mm thick sheets at the same temperature for 5 min using a hydraulic press (Carver, Inc., Wabash, IN, USA) at a pressure of 50 bar. The neat PLA samples were prepared only through compression molding into 1 mm thick sheets for 5 min. at 170 °C and 50 bar pressure of the polymer after drying.

### 2.3. Accelerated Weathering Test

The accelerated weathering of the PLA and PLA/TiO_2_ samples was conducted in an accelerated weathering tester Model QUV/se (Q-LAB, Westlake, OH, USA). The weathering conditions were in accordance with the Cycle-C of the ASTM D4329 standard. Fluorescent lamps (UVA-340) with 0.76 W m^−2^ irradiance (wavelength 340 nm) were used with cycles of 8 h UV irradiation at 50 °C, followed by 4 h dark condensation at 50 °C. These consecutive cycles were applied to the specimens attached to the test panels without any interruption. The effects of the accelerated weathering were investigated for four exposure periods: 0, 500, 1000 and 2000 h. Samples were collected for analysis after each period and they were designated as ‘PLA/*x* h’, where *x* denotes the accelerated weathering period in hours.

### 2.4. Characterization Techniques

#### 2.4.1. Surface Visual Changes

After each accelerated weathering period, visual inspection of variations in colour, gloss, transparency and roughness of the specimens was carried out. The visual inspection was made by comparing photographic images of the unweathered and weathered specimens.

#### 2.4.2. X-ray Diffraction Analyses (XRD)

XRD analyses were performed using an Empyrean (Panalytical, Almelo, The Netherlands) equipped with a Cu K_α_ (λ = 0.1540 nm) source. The generator was operated at 40 mA and 45 kV. Samples were scanned from 5 to 80° at a scanning rate of 0.0130° 2θ.

#### 2.4.3. Atomic-Force Microscopy (AFM)

Detailed information about three-dimensional changes in the surface topography of the PLA surface (neat and nanocomposite) after accelerated weathering degradation was obtained using AFM. An MFP-3D system (Oxford Instruments Asylum Research, Santa Barbara, CA, USA) equipped with an AC160TS cantilever (Al reflex-coated Veeco model, OLTESPA, Olympus, Tokyo, Japan) was used for the image scanning and determination of the mechanical properties by the application of an amplitude modulation-frequency modulation (AM–FM) nano-mechanical tool kit. This mode represents an extension of the standard tapping mode, while the AFM cantilever with tip is excited simultaneously at both its fundamental resonant frequency and another eigenmode. The fundamental resonance allowed the observation of the topographical structures of the sample and tracking of the frequency and the amplitude shift of the other eigenmode was used for an investigation of the mechanical properties in the surface area. The obtained frequency shift Δf was used for an estimation of the interaction stiffness $\Delta k^{FM}$ through Equation (1),

(1) $$\Delta k^{FM}\approx 2k_{c}\frac{\Delta f}{f_{c}}$$

where *k_c_* is the cantilever spring constant, and *f_c_* is the frequency of the cantilever eigenmode. Young’s modulus of the samples was obtained using a general Hertz model describing the contact mechanics between the AFM tip and the sample. A sample standard with known Young’s modulus (31.7 MPa for PLA) was first used for the cantilever calibration by an evaluation of its elasticity. This cantilever elasticity was then used to obtain absolute values of the stiffness and Young’s modulus of the PLA and PLA/TiO_2_ samples on the entire surface area.

#### 2.4.4. Scanning Electron Microscopy (SEM)

SEM analyses of the samples were performed to obtain 2D images of the damaged surfaces using a NanoSEM 450 electron microscope (FEI Nova, Hillsboro, OR, USA), at an accelerating voltage of 2–5 kV. A thin Au layer of a few nanometers thick was sputter-coated onto the surfaces and cross-sections of the samples to obtain high-resolution images and to avoid the accumulation of electrons on the measured layer.

#### 2.4.5. Surface Energy

The changes in surface wettability of the PLA and PLA/TiO_2_ composites caused by accelerated weathering were evaluated by static contact angle (CA) measurements using the sessile drop method. A surface energy analysis system OCA35 (DataPhysics, Filderstadt, Germany) equipped with a CCD camera was used for this purpose. Water, formamide and ethylene glycol were used as testing liquids to evaluate the total surface free energy, as well as the polar and dispersive components, using the conventional Owens–Wendt–Rabel–Kaelble method. A droplet of approximately 3 μL from each testing liquid was placed on the air-facing samples. The CA was calculated after approximately 3 s to allow thermodynamic equilibrium between the liquid and the sample interface to be reached. The reported value for each testing liquid corresponds to the mean of at least five measurements taken on different parts of the substrate surface. The obtained CA values were subsequently used for an evaluation of the total surface energy, as well as its polar and dispersive components.

#### 2.4.6. Gel Permeation Chromatography (GPC)

The number average (*M*_n_) and weight average (*M*_w_) molecular weights and the molar mass dispersity (*Đ_M_*), were determined with GPC/size exclusion chromatography (SEC) equipped with a Wyatt Mini Dawn (Wyatt Technology Corporation, Santa Barbara, CA, USA) multi-angle light scattering detector and a 100 μL Waters Alliance 2695 autoinjector (Waters, Elstree, United Kingdom). The columns were Agilent PL-gel Mixed-D (Agilent, Santa Clara, CA, USA) with a pore size of 5 μm and a particle size of 2 μm, conditioned at 160 °C using an external oven. The flow rate was 1 mL min^−1^, and ultrapure tetrahydrofuran (THF) was used as the mobile phase. PLA samples for analysis were prepared by dissolving a small amount of polymer (35 mg ml^−1^) in THF, followed by vigorous stirring for 30 min. Subsequently, the sample was filtered through a 0.45 mm nylon filter, and then the sample was injected. All the PLA samples with TiO_2_ were dissolved in chloroform and centrifuged to remove excess TiO_2_ to reduce the free TiO_2_ sticking to the column. Chloroform was rotary evaporated before further testing. The resulting solid was dissolved in trichlorobenzene (TCB).

#### 2.4.7. Fourier-Transform Infrared (FTIR) Spectroscopy

FTIR spectroscopy with attenuated total reflectance accessory was used to identify the chemical composition of the biodegradable composites after weathering degradation. An FTIR Frontier spectrometer (PerkinElmer, Waltham, MA, USA) equipped with a ZnSe crystal was used for these analyses, capturing data from 1.66 μm penetration depth, using an average of 8 scans with a resolution of 4 cm^−1^.

#### 2.4.8. Differential Scanning Calorimetry (DSC)

The DSC analyses were performed in a DSC8500 (PerkinElmer, Waltham, MA, USA) differential scanning calorimeter. The PLA samples (5–10 mg) were heated from 30 to 160 °C at 10 °C min^−1^. The melting enthalpies of PLA were determined from the DSC curves and Equation (2) was used to calculate the degrees of crystallinity of PLA, where *ΔH*_m_ is the measured melting enthalpy, Δ*H*_cc_ is the measured cold crystallization enthalpy (which has a negative value because crystallization is exothermic—therefore the use of |Δ*H*_m_| and |Δ*H*_cc_| in the equation and in Table 3), Δ*H*°_m_ is the enthalpy of melting of the 100% crystalline polymer, with a value of 93.6 J g^−1^ for PLA [43], and *W* is the polymer mass fraction in the analyzed sample (1.00 for neat PLA and 0.97 for PLA/TiO_2_ because the mass fraction TiO_2_ was 0.03 in all the nanocomposite samples.

(2) $$X_{c}\left(\%\right)=\frac{\left(\left|\Delta H_{m}|-|\Delta H_{cc}\right|\right)}{W\times \Delta H_{m}^{{}^{\circ}}}\times 100$$

#### 2.4.9. Thermogravimetric Analysis (TGA)

A TGA4000 (PerkinElmer, Waltham, MA, USA) thermogravimetric analyzer (TGA) was used to analyze the thermal degradation behaviour of the samples. The analyses were done from 30 to 600 °C at a heating rate of 10 °C min^−1^ under nitrogen flow (20 mL min^−1^). The sample masses were 10–15 mg.

#### 2.4.10. Tensile Testing

The tensile properties were determined at room temperature in a Lloyd LR 50 k Plus (Lloyd Instruments, Ltd., Fareham, UK) universal testing machine at a stretching speed of 10 mm min^−1^ (ASTM D638). The gauge length was 25 mm, and the sample (dumbbell shape) width and thickness were 3.25 and 1 mm, respectively.

## 3. Results and Discussion

### 3.1. Surface Morphology

**Figure 1:**
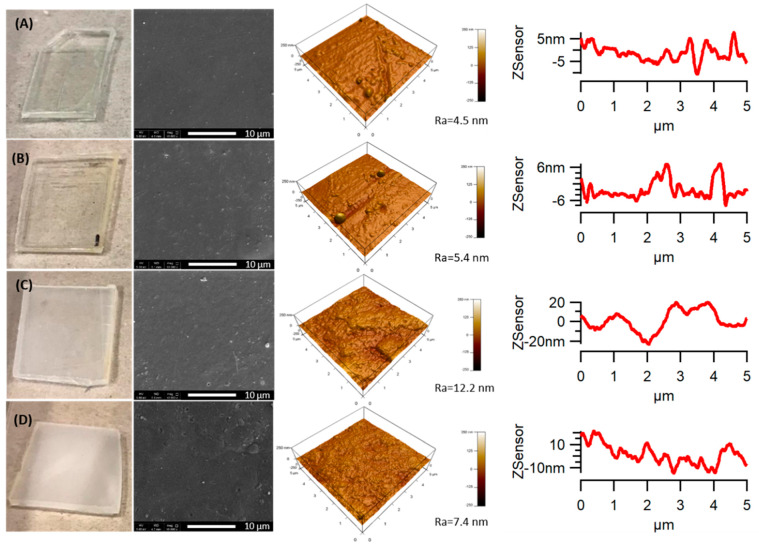
Scanning electron microscopy (SEM) and atomic force microscopy (AFM) images (3D height and line profile) of poly(lactic acid) (PLA) samples before and after accelerated weathering: (**A**) 0 h; (**B**) 500 h; (**C**) 1000 h and (**D**) 2000 h. Note: Ra represents the roughness parameter.

Changes in the color, gloss, transparency and smoothness of the weathered specimens were first visually evaluated by digital photographs. It is seen in Figure 1A that the neat PLA appears totally transparent before weathering because the very fast cooling rate during the molding process did not allow the PLA chains to have conformational ability for crystallization, which gave rise to a completely amorphous structure [34,38]. After 1000 h of accelerated weathering exposure, PLA became less transparent and acquired some opacity. An increase in this whitening was observed with increasing weathering time [38,40]. It means that during the accelerated weathering test, the temperature, moisture and UV-irradiation were enough to initiate the crystallization of PLA [34]. This result is expected because of chain scission during degradation, where the shorter polymer chains could reorganize, forming crystalline domains [40], as will be seen from the XRD and DSC results.

**Figure 2:**
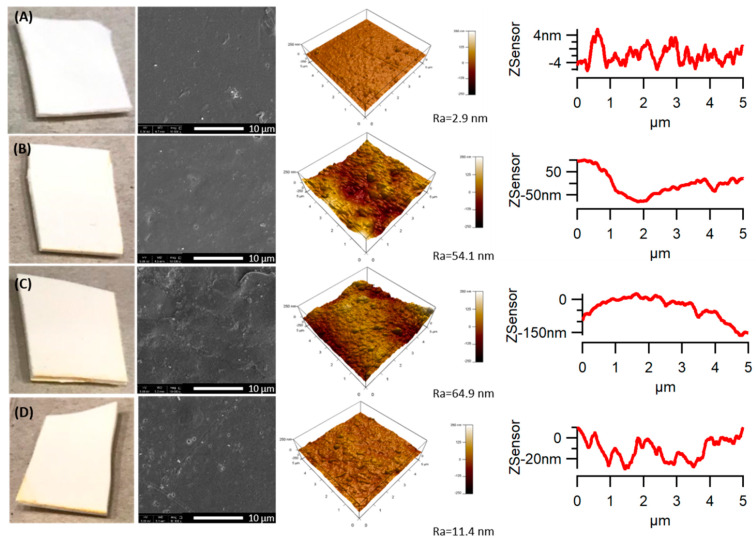
SEM and AFM images of PLA/TiO_2_ samples before and after accelerated weathering: (**A**) 0 h; (**B**) 500 h; (**C**) 1000 h and (**D**) 2000 h. Note: Ra represents the roughness parameter.

The addition of 3 wt % of TiO_2_ in the PLA matrix made the same evaluation impossible because the TiO_2_ particles are white (Figure 2). However, an increase in the yellowness level of the specimens was observed with increasing weathering time [44], changing the white colour of the samples to a pale yellow. The colour change of the samples can be explained by the formation of chromophore groups (e.g., carboxyl, carbonyl, ketone, aldehyde, hydroxyl, ester, etc.) and conjugated double bonds in the polymers by the photo-oxidative reactions during the accelerated weathering exposure [45].

A loss in the glossy character in the PLA/TiO_2_ nanocomposite was also noticed after accelerated weathering. The neat PLA, however, exhibited the same high gloss over the weathering time. The significant colour and gloss change for the PLA/TiO_2_ implies that severe degradation occurred for the nanocomposite, showing that the addition of TiO_2_ nanoparticles accelerated the PLA degradation.

PLA and the PLA/TiO_2_ nanocomposite showed a smooth surface during the accelerated weathering tests when they were visually observed through naked eyes. However, the morphology of the samples during the degradation studies was also investigated through SEM. Figure 1 and Figure 2 show the SEM micrographs taken from the sample surface of PLA and the PLA/TiO_2_ nanocomposite before and after being subjected to accelerated weathering for 500, 1000 and 2000 h. The neat PLA did not show significant changes on the surface after 500 h of weathering time (Figure 1B). However, the surface roughness (Ra) progressively increased up to 1000 h and small holes formed on the surfaces of the PLA samples. With increasing weathering time, more holes formed (Figure 1). The PLA/TiO_2_ nanocomposite showed apparent changes from 500 h under accelerated weathering conditions (Figure 2B). The surface texture of the nanocomposite changed more significantly after 1000 h, and a large number of irregular cavities appeared, while only a few small cavities appeared on the surface of the neat PLA during the same period. The weathering degradation of the PLA/TiO_2_ nanocomposite was faster than that of neat PLA, showing a more severe degradation of the polymer for the same conditions. Adding TiO_2_ to PLA is, therefore, an effective method to enhance the degradation of PLA.

The presence of cracks, cavities and holes present on the sample surface enhance all the degradation processes, because the bulk of the samples will be exposed to UV radiation and will also allow water penetration.

### 3.2. Surface Mechanical Properties

**Figure 3:**
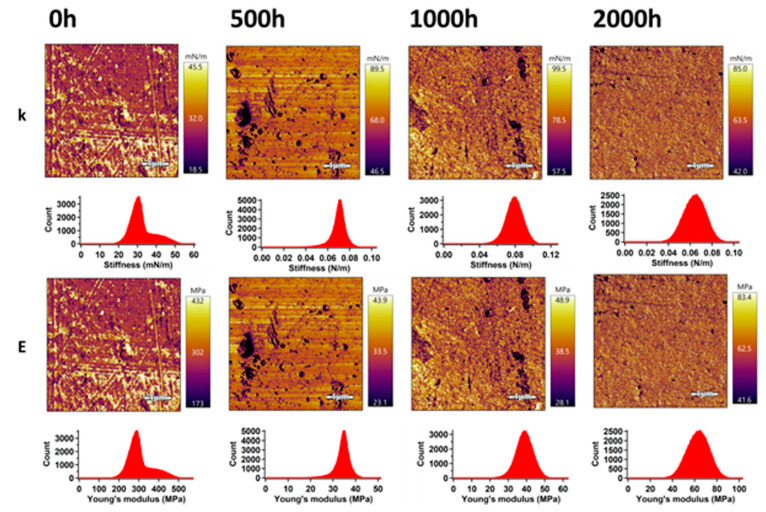
AM–FM images and histograms of PLA samples before and after accelerated weathering; k and E represent stiffness and Young’s modulus, respectively.

**Figure 4:**
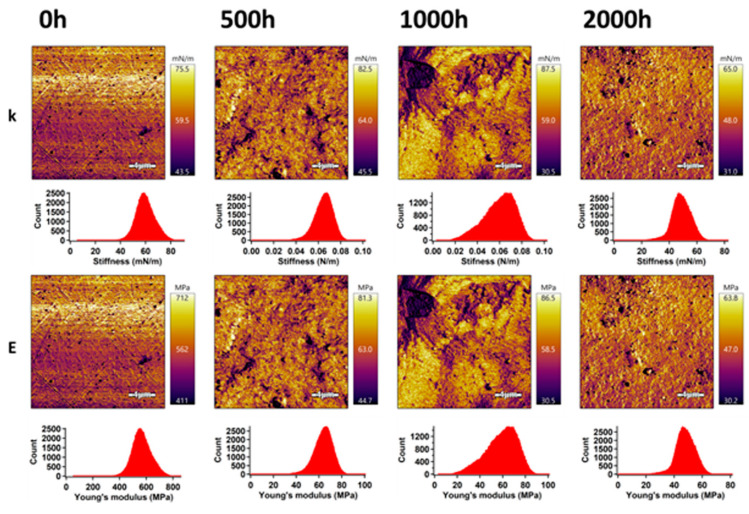
AM–FM images and histograms of PLA/TiO_2_ samples before and after accelerated weathering; k and E represent stiffness and Young’s modulus, respectively.

The mechanical properties of PLA and the PLA/TiO_2_ nanocomposite in the surface area were analyzed by an advanced AM–FM technique using AFM. This technique allows obtaining images with the distribution of stiffness and Young’s modulus in an entire surface area. The results for PLA and the PLA/TiO_2_ samples before and after accelerated weathering exposure are shown in Figure 3 and Figure 4. The mean values of stiffness and Young’s modulus were evaluated using Gaussian fitting of the most occurred peaks in the related histograms, and these are summarized in Table 1.

The PLA samples exhibited two peaks in the stiffness and Young’s modulus histograms. The stiffness and Young’s modulus of the phase with the highest intensity showed values of 29.7 mN m^−1^ and 29.5 MPa, respectively. Five hundred hours of weathering had a significant effect on an increase in the surface mechanical properties, while the stiffness and Young’s modulus showed values of 70.5 mN m^−1^ and 34.6 MPa, respectively. In addition, the two phases merged, and just one narrow peak was observed. An ageing time of 1000 h led to a continuing increase in mechanical properties. However, 2000 h of ageing caused a slight decrease in the stiffness despite the increase in Young’s modulus (note that these are surface properties that are different from the Young’s modulus of the bulk sample, which is directly related to sample stiffness). Approximately double values compared to those of the unweathered PLA samples were achieved. These results suggest a higher degree of crystallinity for PLA after weathering, corroborating with the DSC analysis. The broader peak observed should be related to changes in molecular weight and higher *Đ_M_* exhibited during weathering, as further discussed in the GPC section.

A similar trend was observed for the PLA/TiO_2_ samples during the first 1000 h of ageing, but with less apparent changes. The presence of TiO_2_ in the PLA/TiO_2_ samples was responsible for higher values of stiffness and Young’s modulus compared to the PLA. Stiffness and Young’s modulus of the unweathered PLA/TiO_2_ samples achieved values of 59.0 mN m^−1^ and 58 MPa, respectively. The mechanical properties in the surface area after 500 h of weathering time was only slightly enhanced, while stiffness and Young’s modulus achieved values of 65.3 mN m^−1^ and 64.3 MPa, respectively. After 1000 h of weathering time, the surface mechanical properties of PLA/TiO_2_ were slightly deteriorated and after 2000 h, the values were even lower at 48.5 mN m^−1^ and 47.8 MPa for stiffness and Young’s modulus, respectively. This behaviour is supported by the degree of crystallinity, as observed by DSC. In addition, lower values of stiffness and Young’s modulus of PLA/TiO_2_ were found in comparison with PLA samples aged at the same weathering exposure time. These mechanical changes in the surface area after accelerated weathering proved the lower stability of the PLA/TiO_2_ over the weathering time (2000 h). The extent of degradation was more significant for the PLA samples with TiO_2_ nanoparticles, showing that the addition of TiO_2_ accelerated the degradation process of the PLA/TiO_2_ samples in the surface area.

### 3.3. XRD Studies

**Figure 5:**
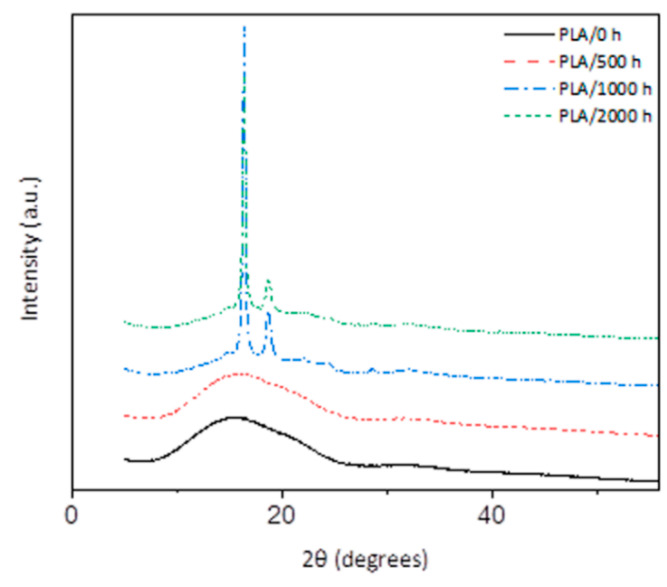
X-ray diffraction spectra of neat PLA at different accelerated weathering times.

XRD provides an ideal method to monitor changes in the crystallization of polymers during degradation. The X-ray diffraction patterns of PLA at different accelerated weathering times are compared in Figure 5. The unweathered PLA appears totally amorphous without any recognizable peaks. The same is observed after 500 h of accelerated weathering exposure, which is in line with the transparent visual appearance of the sample. After 1000 h, a change in transparency was observed, and diffraction peaks appear at 2θ = 16.5° and 18.7°, which correspond to the (110/200) and (203) planes from the crystalline structure of PLA [32,46,47]. The result confirms the formation of a crystal structure during the degradation of neat PLA. It is known that the amorphous regions are the first phase being attacked during UV and hydrolysis degradation, followed by the crystalline phase [46].

**Figure 6:**
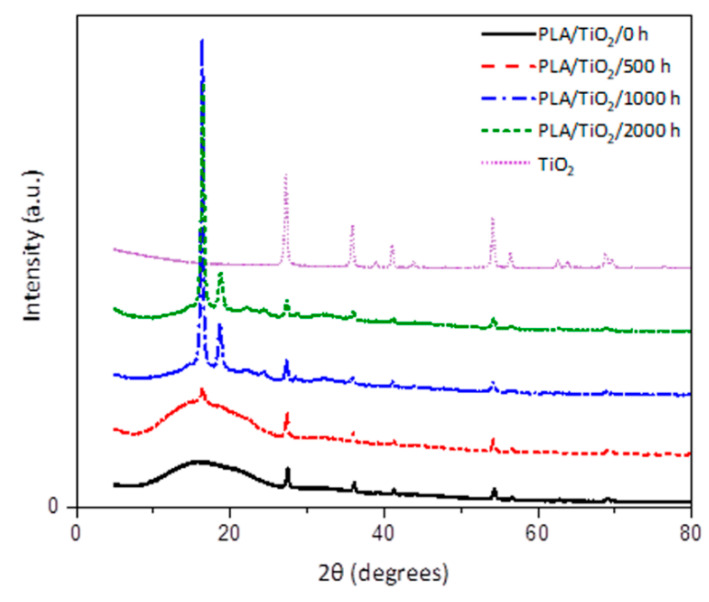
X-ray diffraction spectra of PLA/TiO_2_ nanocomposite at different accelerated weathering times.

The XRD profiles of the PLA/TiO_2_ nanocomposite after weathering degradation are shown in Figure 6. After 500 h of weathering exposure, there is a slight indication of crystallinity starting to develop as a result of weathering. In addition to the broad diffraction peak centered at 16.5°, which can be assigned to neat PLA, crystalline peaks are also observed at 2θ values of 27.4°, 36.1°, 39.2°, 41.2°, 44.0°, 54.2° and 56.6°, which correspond to the (110), (101), (200), (111), (201) and (220) planes of the TiO_2_ nanoparticles, as confirmed in the XRD from neat rutile–TiO_2_ [32,48]. The higher mobility of the shorter chains formed during degradation allows for the formation of a crystalline phase, which increases the crystallinity [49]. The deterioration of the crystalline phase after 2000 h of weathering, as was observed in other analyses, is not so obvious from the diffractograms in Figure 6.

The most important observation from the diffractograms in Figure 5 and Figure 6 is that crystallinity already started developing in PLA/TiO_2_ during the first 500 h of weathering. This was not observed for the neat PLA, which suggests (in line with the other results) that the TiO_2_ to some extent accelerates the weathering degradation of PLA.

### 3.4. Surface Wettability

**Figure 7:**
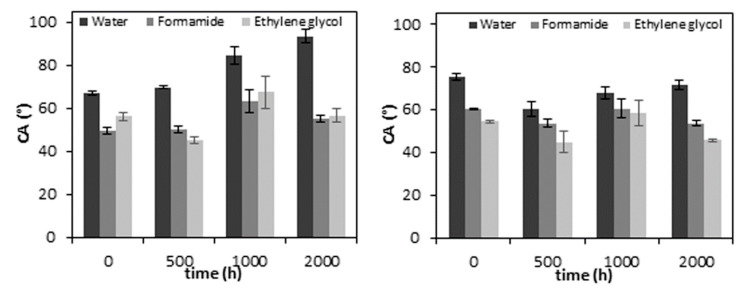
CAs of water, formamide and ethylene glycol in neat PLA (left) and PLA/TiO_2_ (right) as a function of increasing weathering exposure time.

Wettability analysis is necessary for an understanding of the degradation behaviour on the surface of the sample. Figure 7 shows the CA results of neat PLA and the PLA/TiO_2_ nanocomposite as a function of the accelerated weathering time. The CA measurements are shown for water, formamide and ethylene glycol for neat PLA and the PLA/TiO_2_ nanocomposite.

**Figure 8:**
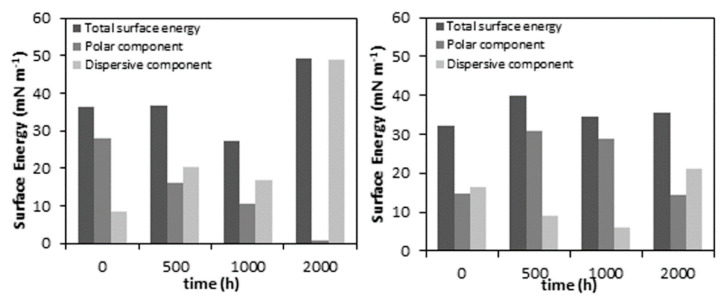
Surface energy of neat PLA (left) and PLA/TiO_2_ (right) as a function of increasing weathering exposure time.

The neat PLA samples show a significant change with increasing accelerated weathering exposure. The data reported in Figure 7 shows that the accelerated weathering causes an increase in the water CA, thus the hydrophobicity of the neat PLA increases. PLA is naturally more hydrophobic due to its methyl side groups, but it also contains polar oxygen linkages, which confer hydrophilic properties to the polymer [50,51]. The increase in water CA and decrease in surface free energy, particularly the polar component, confirm that the accelerated weathering degradation generates hydrophobic surface properties on PLA within a relatively short exposure time, which can be attributed to several factors such as chemical and physical changes. Some authors suggested an expected decrease in water contact angle because the cleavage of ester linkages, which produces lower M_w_ substances (e.g., lactic acid oligomers and monomer) containing hydrophilic end groups such as carboxylic and hydroxyl groups on the polymer chains [50,51,52,53]. However, as was well reported, shorter polymer chains are easily able to reorganize into crystalline domains. Erosion of the amorphous bulk and reorganization of polymer chains increase the extent of crystallinity, resulting in a closed surface for water absorption. In addition, changes in the hydrophobic properties of the PLA can also be associated with an increase in the CH_3_ density in the remaining chains during surface erosion [50,51,52,53]. The proposed change in the density of the polar and non-polar phases is corroborated with a decrease in the surface polar component of the total surface energy, as shown in Figure 8.

In addition to this, physical changes were also observed during degradation, even as an increase in roughness. This behaviour is found in the neat PLA and PLA/TiO_2_ nanocomposite in the SEM and AFM analyses, and this can also contribute to an increase in hydrophobicity [54]. However, this is not the only reason to change the water CA of the PLA/TiO_2_ nanocomposite over time. It is therefore suggested that the hydrophobic behaviour of the degraded PLA samples was the result of the factors described in the previous paragraph.

The water CA of the PLA/TiO_2_/0 h (75.0° ± 1.5°) sample is slightly higher than that of the neat PLA/0 h (66.9° ± 0.8°). This is probably because the sample surface of neat PLA was smoother than the nanocomposite surface, while the hydrophilicity of the TiO_2_ nanoparticles probably also contributed to this change in CA. The addition of nanofillers to the PLA matrix led to a rougher surface. Current works [4,7] show that low concentrations of nano TiO_2_ can be well dispersed in PLA. When the nanoparticles are well distributed in the polymer matrix, such a roughness morphology can be observed on the surface, contributing to some increase in the CA [55]. A similar trend was observed in the water CAs measured over the degradation time of the PLA/TiO_2_ samples. After 500 h of weathering exposure, the values of CA were lower than those of the unweathered samples. After 1000 h, they slightly increased, but showed lower values than that for PLA/TiO_2_/0 h. The hydrophilicity of the PLA/TiO_2_ nanocomposite has been attributed to the hydrophilicity of the filler, which exhibits easier contact and permeability of water into the polymer matrix [51]. Both the physical and chemical properties of the PLA/TiO_2_ nanocomposite allow similar values of water CA after 2000 h of accelerated weathering exposure, hiding any changes that may have been the result of oxidative degradation.

### 3.5. Molecular Weight (GPC)

To confirm chain scission and changes in the molecular weight distribution of the polymer, gel permeation chromatography was performed for samples exposed to 0, 1000 and 2000 h in accelerated weathering conditions. Table 2 shows the changes in the molecular weight of PLA and the PLA/TiO_2_ nanocomposites as a function of accelerated weathering time, and also reports the percentage of degradation and *Đ_M_*.

The initial *M*_w_ of PLA decreased from 89.5 to 9.0 kg mol^−1^ after 2000 h of artificial weathering. The *M*_w_ of PLA/TiO_2_ showed a smaller decrease from 44.0 to 15.0 kg mol^−1^ after 2000 h under the same conditions. In agreement with prior works, it is necessary to mention that the major difference between the unweathered PLA and PLA/TiO_2_ was because of the blending and the fabrication of the samples at high temperatures [23,39]. Since *M*_n_ and *M*_w_ were different for neat PLA and PLA in PLA/TiO_2_ at the start of the weathering degradation, we added the percentages of degradation into Table 2 in an effort to obtain a more valid comparison. Unfortunately, the results are still questionable and therefore we are not going to try and draw any specific conclusions from the trends observed in Table 2.

It is, however, obvious that the *Đ_M_* of PLA remained relatively stable at 1.3. This strongly suggests that the chain scissions randomly occurred in all the molecules of PLA [23,39]. However, there is a difference between the *Đ_M_* of neat PLA and PLA in PLA/TiO_2_, which suggests that the kinetics of chain cleavage was different. This, as well as the other *M*_W_ results, to a large extent confirm the strong influence of TiO_2_ in accelerating the degradation of PLA. This is, for example, clear from the observation that after only 10 min at a relatively low mixing temperature of 170 °C, the *M*_w_ of PLA was reduced by more than 50%.

### 3.6. Surface Chemistry (FTIR)

**Figure 9:**
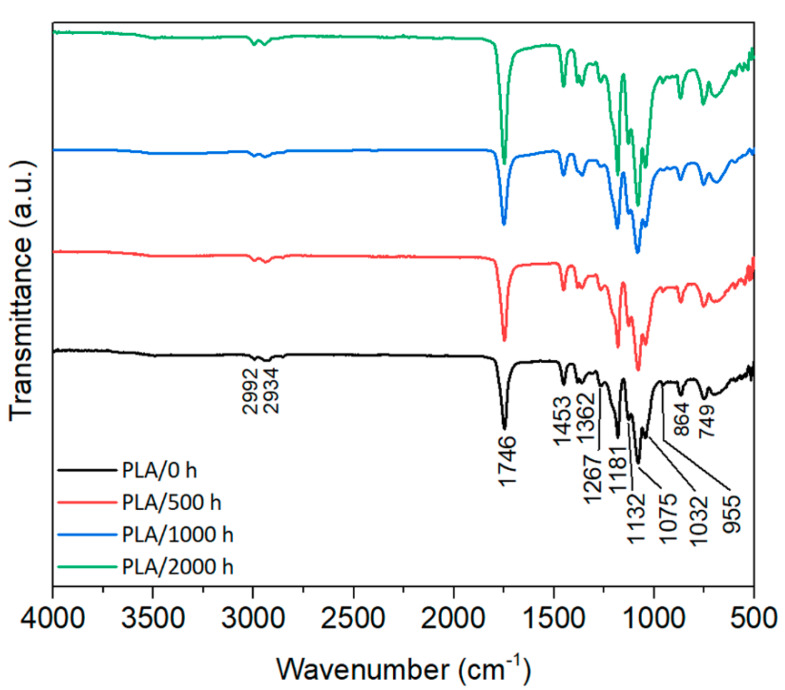
FTIR spectra of neat PLA before (0 h) and after (500, 1000 and 2000 h) accelerated weathering.

The infrared spectra of PLA (Figure 9) present the characteristic bands of the neat polymer identified in previous works [17,40,44,56,57], i.e., a broadband centered at 3200 cm^−1^ attributed to the OH group from carboxylic acid, the region of 2800–2900 cm^−1^ assigned to asymmetric and symmetric methylene chains (–CH_2_–), and the strong absorbance band at 1748 cm^−1^ attributed to the stretching vibration of the amorphous carbonyl groups. The other observed bands, positioned at 1452, 1382 and 1360 cm^−1^, are due to the CH_3_ asymmetric and symmetric deformations. The C–O stretching characteristic bands are seen at 1268 and 1080 cm^−1^. Crystalline domains are indicated by 1210 and 956 cm^−1^ from the C–O stretching, while the amorphous phase is indicated by the 1180 and 1043 cm^−1^ peaks.

Accelerated weathering deteriorates the structure of PLA via polymer cleavage leading to chain scission, especially at the C–O (observed at 1262 cm^−1^ and 1180 cm^−1^) and C–C (866 cm^−1^) ester bonds from the PLA backbone structure. Some authors [40,44] discussed that the infrared bands after degradation change intensities or shift to lower wavenumbers. However, no significant changes were detected after degradation of neat PLA, and no vibration modes were suppressed or appeared due to degradation.

**Figure 10:**
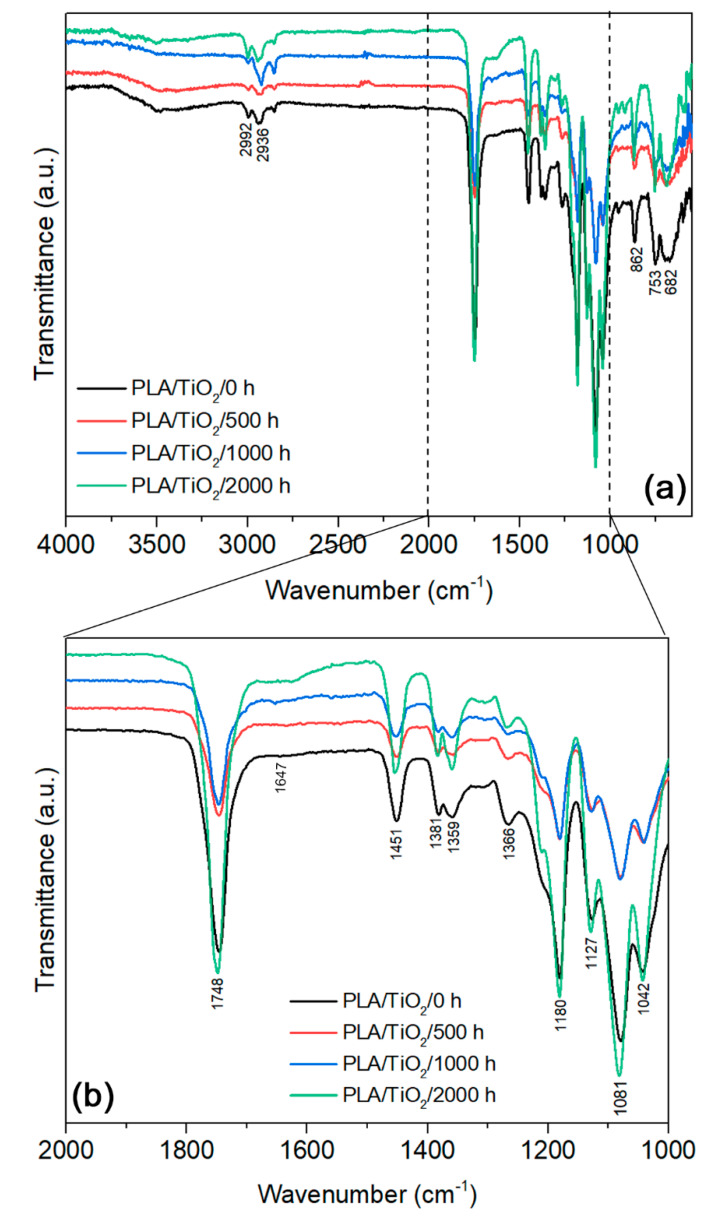
**(a)** FTIR spectra and the characteristic transmittance peaks of PLA/TiO_2_ before (0 h) and after (500, 1000 and 2000 h) accelerated weathering. (**b**) Detailed area: C=C shoulder at 1647 cm^−1^.

Figure 10 shows the FTIR spectrum of the PLA/TiO_2_ samples and the characteristic transmittance peaks after different periods of accelerated weathering. Similar behaviour to that of PLA was observed, showing that the nanocomposite followed the same degradation process than neat PLA.

The photodegradation of PLA occurs according to a Norrish II mechanism of carbonyl polyester. This mechanism involves chain cleavage and the presence of hydroperoxide (OH) and C=C double bond at the newly formed chains [34,44,58]. After 2000 h of accelerated weathering, this was observed in the PLA/TiO_2_ sample (Figure 10). PLA/TiO_2_/2000 h spectra clearly exhibits a shoulder at 1647 cm^−1^, which reveals the generation of a new C=C bond that proves that the photodegradation via a Norrish II type mechanism was improved by the presence of rutile–TiO_2_.

### 3.7. Thermal Properties

**Figure 11:**
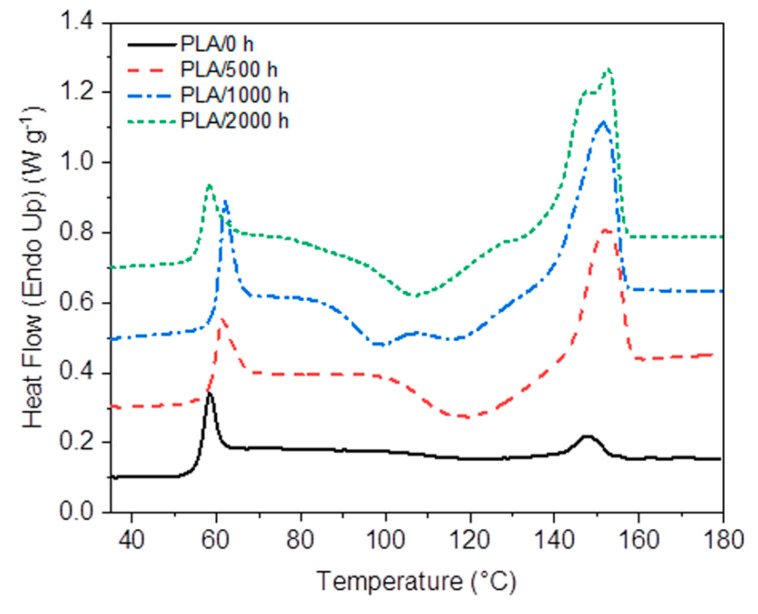
DSC first heating curves of neat PLA after accelerated weathering degradation.

**Figure 12:**
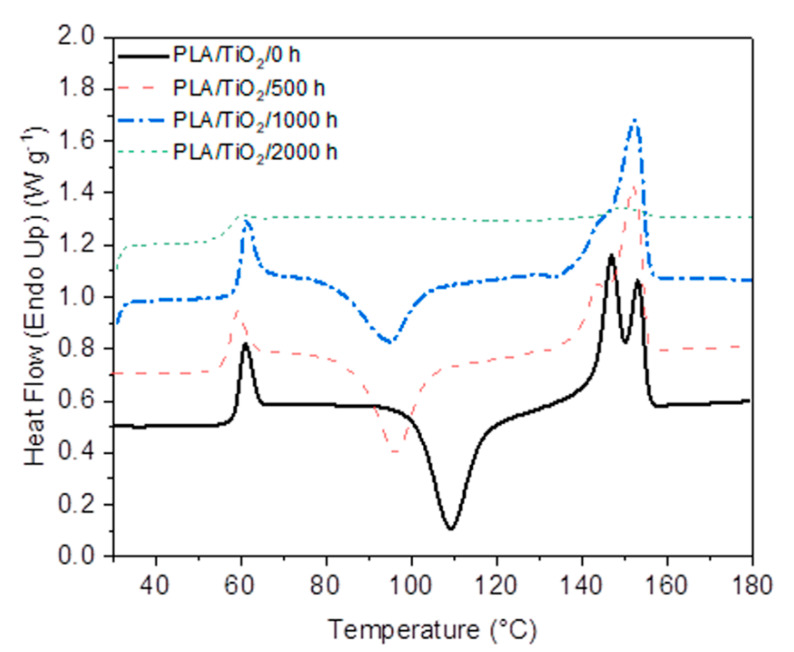
DSC first heating curves of PLA/TiO_2_ nanocomposite after accelerated weathering degradation.

DSC was selected to study the thermal properties of the PLA samples, neat and the nanocomposite. Figure 11 and Figure 12 show the first heating curves of PLA and the PLA/TiO_2_ nanocomposite, before and after different accelerated weathering exposure times. The related data from the DSC analysis (melting and cold crystallization enthalpy and temperature, glass transition and degree of crystallinity, respectively, Δ*H*_m_, Δ*H*_cc_, *T*_m_, *T*_cc_, *T*_g_ and *X*_c_) are summarized in Table 3. Similar to the results reported in the literature [8,56,59], neat PLA shows a glass transition at 57 °C, a broad shallow cold crystallization peak around 120 °C and a small melting peak at 148 °C in the heating curve, while the absence of a crystallization peak in the cooling curve (not shown) implies that the initial crystal content in this sample was quasi-inexistent. This behaviour was expected because PLA was completely transparent before ageing, indicating an amorphous structure. In addition, the fact that the cold crystallization and melting enthalpies were approximately the same, and the absence of any crystallinity peaks in the XRD analysis, supported our observation.

After accelerated weathering, an increase in the melting enthalpy of PLA is observed in Table 3. The cold crystallization also shows a much more pronounced exotherm with both the cold crystallization and melting enthalpies increasing, and with the *T*_CC_ decreasing, with increasing weathering time. The observed behaviour during cold crystallization was probably the result of the easier crystallization of the shorter chains, formed after chain scission by photo and hydrolysis degradation [2,38,49]. As the weathering time increased, the difference between the melting and cold crystallization enthalpies increased, indicating that there must have been a fair amount of crystallinity in the samples before DSC analysis since these values were taken from the first heating curves. This shows that some crystallization already occurred during the weathering process, especially for long weathering times. This was visually observed through the samples becoming translucent, and through the appearance of crystallinity peaks in the XRD spectra for the 1000 and 2000 h UV exposed samples. Table 3 shows inconsistent changes in the glass transition with increasing weathering time, confirming a complex influence of factors like changes in free volume and the configuration of the amorphous phase on this parameter [12,46,49,58,60,61]. In addition, two melting peaks were observed at 148 and 153 °C for PLA/2000 h, which could reflect the melting of β and α crystallites, that have different melting temperatures due to different size and perfection [62,63,64]. The double-peak could also have been the result of a melting–recrystallization–melting process, or of the melting of different sized lamellae formed during the degradation process [65].

The glass transition of PLA/TiO_2_ before weathering is observed at 60 °C, while a second melting peak started to develop with increasing weathering exposure time. The unweathered sample shows a well-developed double melting peak at 147 and 153 °C, which can be explained as the melting of α- and β-crystals, or as a melting–recrystallization–melting phenomenon. Since the lower temperature peak reduced in size and eventually disappeared with increasing weathering time, the latter explanation is probably the more correct one. When comparing the respective DSC curves in Figure 11 and Figure 12, and the enthalpy values in Table 3, it is clear that the titania nanoparticles acted as nucleation centers for the cold crystallization of PLA [62,63,64]. The cold crystallization peaks in Figure 12 are much more intense and well resolved than those in Figure 11, and the crystallinity values are observably higher for the 500 and 1000 h weathered samples. As already discussed earlier in this study, higher crystallinities is an indication of the formation and recrystallization of shorter chains formed during degradative chain scission. This also points to the catalytic effect of the TiO_2_ on the UV-initiated degradation of PLA, which is particularly evident when one looks at the DSC curve of the 2000 h weathered sample in Figure 12, where an almost non-existent cold crystallization and melting were observed. This observation can be explained as such an extensive weathering degradation that the chain segments after UV-initiated scission became too short to properly arrange into crystallites. As in the case of neat PLA, no crystallization peak was observed upon cooling for all the PLA/TiO_2_ samples.

In conclusion, the degradation of the PLA started in the amorphous structures, transforming them into crystalline domains. Thus, the polymer chains from chain cleavage during UV and hydrolysis degradation were of lower molecular weight. These shorter chains had a higher mobility to re-organize and create crystalline domains in the amorphous matrix, giving rise to a semi-crystalline structure. This crystallinity increased with the exposure time in accelerated weathering. However, the crystallinity decreased when the degradation extended into the crystalline domains once the degradation of the amorphous region was over [2,23,34,46,49,56,62,66], which was observed in the PLA/TiO_2_/2000 h sample. This is in line with most of the other results, showing that the presence of TiO_2_ accelerated the weathering degradation of PLA, in addition to promoting the cold crystallization ability of PLA.

### 3.8. Thermogravimetric Analysis (TGA)

**Figure 13:**
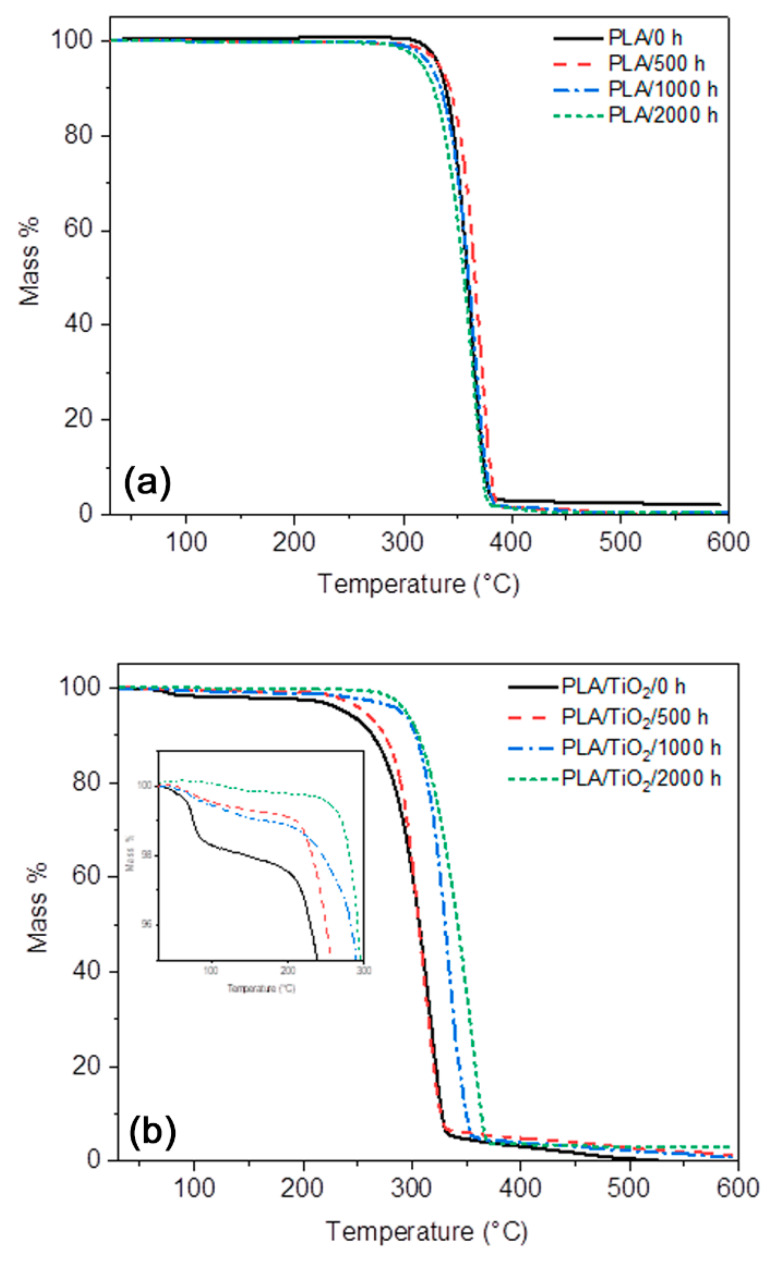
TGA curves for the (**a**) PLA and (**b**) PLA/TiO_2_ samples after different periods of accelerated weathering.

Figure 13 and Table 4, respectively, show the TGA curves, the maximum decomposition temperatures, and the decomposition temperatures at 5% and 50% of mass loss for neat PLA and the PLA/TiO_2_ nanocomposite for different periods of artificial weathering. Only one degradation step is observed for the neat polymer and the nanocomposite. However, before the significant mass loss step that corresponds to the decomposition of the PLA, the TGA curves of the PLA/TiO_2_ samples present an initial mass loss from room temperature to 100 °C attributed to the loss of moisture. This was also observed by other authors [67], and is probably the result of the more hydrophilic nature of the TiO_2_ nanofiller [51]. A single-step degradation process is a good indicator that degradation was homogeneous along the thickness of the specimen [37]. This is supported by the homogeneous surface degradation of the sample in the AFM and SEM images.

It should be noted that the thermal stability is better for the neat PLA than for the PLA/TiO_2_ nanocomposite. However, some works report a faster degradation rate for neat PLA than for the PLA/TiO_2_ and attribute it to the TiO_2_ particles acting as heat barriers in the early stages of thermal decomposition, which improves the thermal stability of the nanocomposite [5,61]. This behaviour was not observed in our study. It is highly probable that the moisture in the PLA/TiO_2_ samples took part in the degradation process at elevated temperatures, which accelerated the thermal and hydrolysis degradation that co-occur [56].

Neat PLA has an initial decomposition temperature around 300 °C, and a significant mass loss is observed between 300 and 383 °C, which corresponds to the decomposition of the PLA. This involves an intramolecular transesterification leading to the formation of lactide and cyclic oligomers, followed by the formation of acrylic acid from cis-elimination as well as carbon oxides and acetaldehyde from fragmentation reactions [5]. After this, only a gradual mass loss is observed until a constant mass is reached. The degradation temperatures corresponding to 5%, 50% and maximum mass loss of PLA were found to be 332, 359 and 362 °C. In all the samples, similar TGA curves were observed for PLA before and after weathering. However, a slight shift to higher temperature values was noticed in PLA/500 h, which indicates an increase in polymer stability as a result of the plasticizing effect of the oligomeric products generated during degradation of the PLA [68]. For PLA/2000 h, these temperatures decreased again owing to weathering degradation.

**Figure 14:**
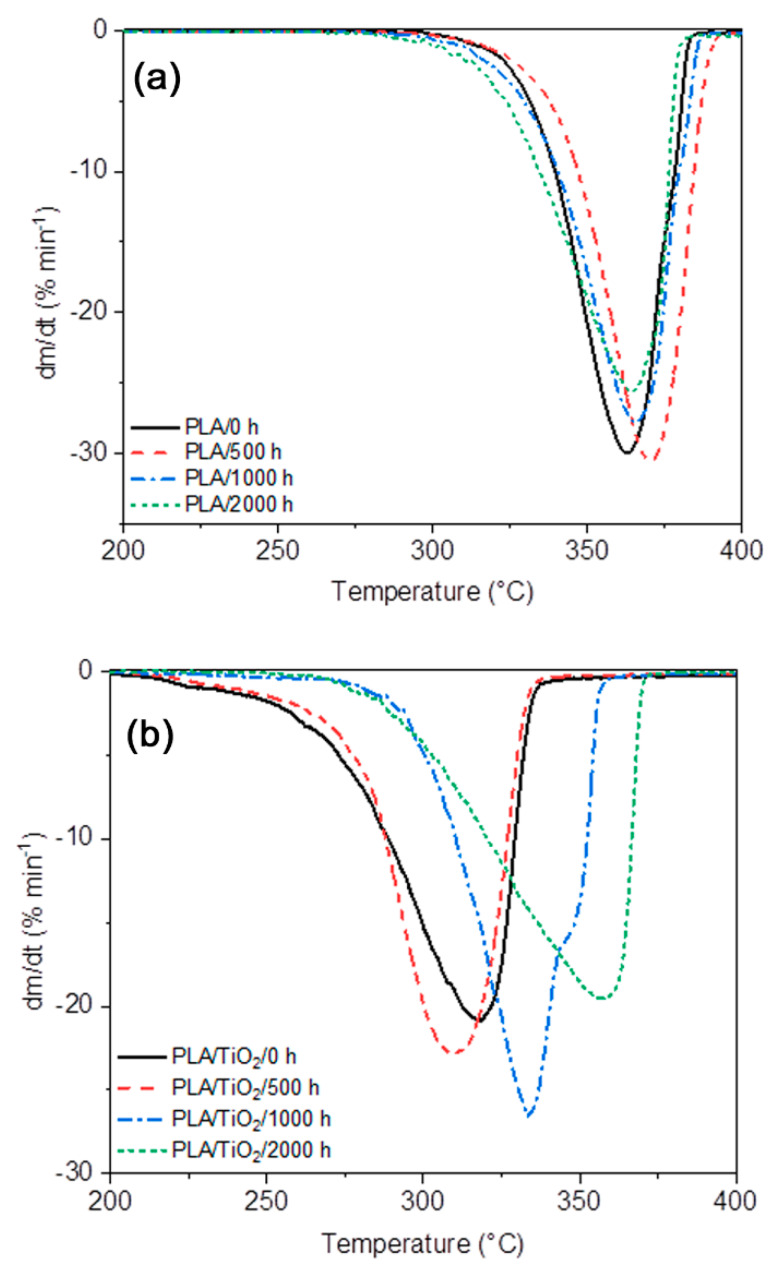
Differential TGA curves for the (**a**) PLA and (**b**) PLA/TiO_2_ samples after different periods of accelerated weathering.

In the case of the PLA/TiO_2_ nanocomposite, the initial decomposition temperature shifted to lower temperatures, between 200 and 300 °C, when TiO_2_ was present. It is known that the hydrolytic degradation mechanism is dominant up to 215 °C [5], and that the moisture present in the PLA/TiO_2_ nanocomposite is the reason for this. After this, the significant mass loss up to 325 °C corresponds to the PLA decomposition in the PLA/TiO_2_ nanocomposite. Finally, the constant mass remaining at the end of each TGA experiment corresponds to the inorganic material, i.e., the TiO_2_, which is very close to the theoretical mass of TiO_2_ in the nanocomposite. The curves in Figure 14 show that the accelerated weathering of the PLA/TiO_2_ nanocomposite caused the degradation to start at slightly higher temperatures. The degradation temperatures at 5% and 50% of mass loss, that are presented in Table 4, show an increase over the weathering time from 239 to 295 and 307 to 342 °C, respectively. The same shift towards a higher *T*_max_ value from 318 to 357 °C indicates an increase in polymer stability after this weathering time.

The PLA/TiO_2_ samples, after weathering exposure, had lower *M*_w_ values, as discussed before, but they were also organized in crystalline domains that degraded more slowly during TGA analysis [12,58]. Another explanation for this result is the proportion of TiO_2_ in the yield samples after weathering. This is higher, and the TiO_2_ nanoparticles could improve the thermal stability of the PLA, acting as thermal blockers against heat diffusion [69]. As expected from previous analyses, the PLA/TiO_2_ after 2000 h accelerated weathering are much more damaged than the neat PLA/2000 h, because the TiO_2_ accelerated the weathering degradation. Therefore, the nanocomposite had a slightly lower thermal stability than the neat PLA. The characteristic degradation temperatures at 5% and 50% mass loss were, respectively 317 and 356 °C for PLA/2000 h and 295 and 342 °C for PLA/TiO_2_/2000 h (Table 4).

### 3.9. Mechanical Properties

**Figure 15:**
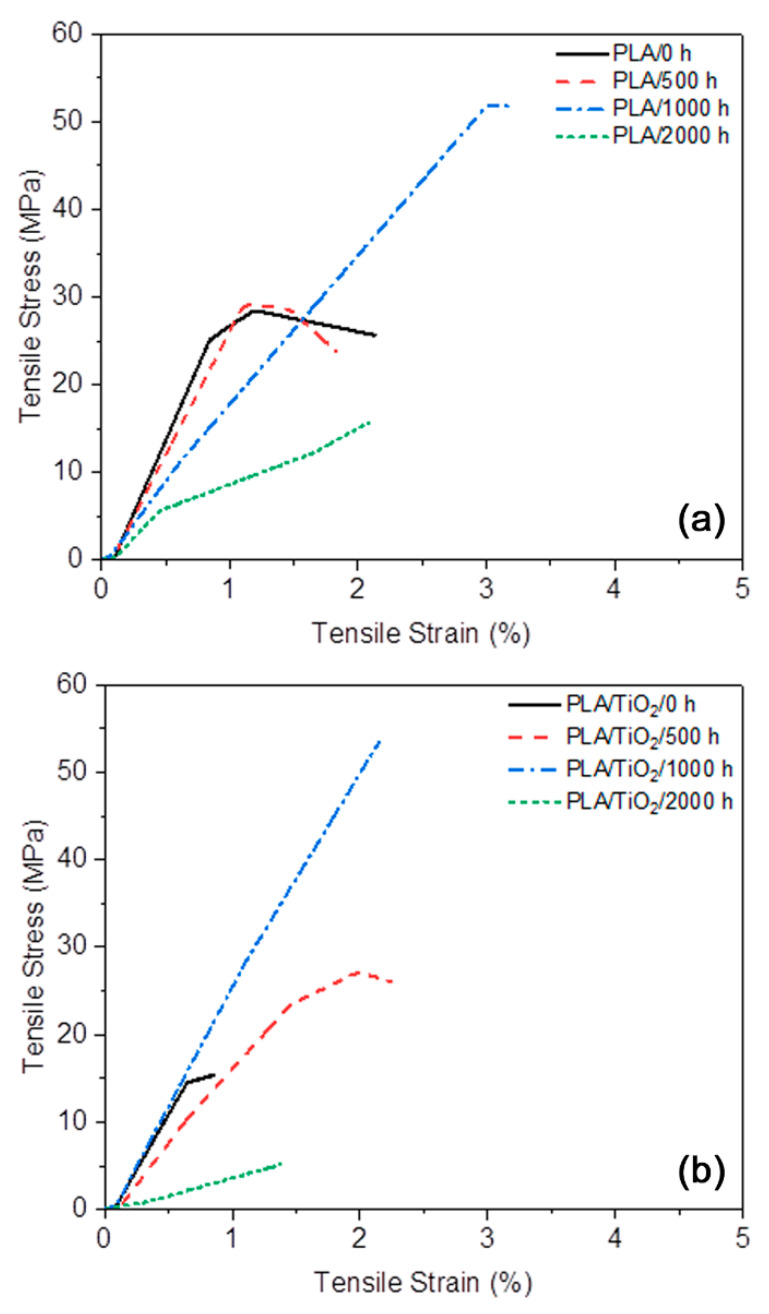
Representative tensile stress–strain curves of (**a**) PLA and (**b**) PLA/TiO_2_ after different periods of accelerated weathering.

Table 5 summarizes the tensile properties of the neat PLA and PLA/TiO_2_ nanocomposite after different periods of accelerated weathering exposure times. Figure 15 presents an example of each stress–stain curve of all the studied samples. It is seen in Table 5 and Figure 15 that after 500 h of accelerated weathering, similar strength (σ_break_) and elongation (ε) were observed for the PLA/0 h and PLA/500 h samples. After longer periods of accelerated weathering, there was an exponential increase in strength and elongation. It is clear that the weathering provided toughness to the PLA samples up to 1000 h of exposure. Chain scission and re-organization of the polymer chains during the degradation process increased the crystallinity of the PLA, as was observed from the DSC and XRD analysis results. These changes improved the tensile properties of the PLA, showing higher strength and strain [34,40,69]. However, it was also expected that the Young’s modulus (E) would increase [40], but this was not observed in our study. According to the literature [70], a finer structure of crystallites will lead to a more ductile material. In contrast, larger crystallites will cause the polymer to be stiffer—even if the degree of crystallinity were the same—so that this morphology could be the reason for the observed results. The increase in the crystallinity of the PLA/2000 h (observed in DSC) was not sufficient to maintain the tensile properties of the PLA, and the chain scission resulted in a significantly lower strength value for this sample [34,40,69].

The tensile strength results for the PLA/TiO_2_ samples were not significantly different from those of the neat PLA samples (Table 5 and Figure 15). The PLA/TiO_2_ nanocomposite showed slightly lower mechanical properties than neat PLA before and after weathering, except for the 1000 h exposed sample, because the presence of the nanofiller constrained the mobility of the PLA molecular chains [5,34]. As with neat PLA, there was significant deterioration of the tensile strength and the elongation at break after 2000 h of weathering exposure. However, the PLA/TiO_2_ nanocomposite showed the best mechanical properties after 1000 h of accelerated weathering, which time period also corresponded with the higher degree of crystallinity observed in the DSC analysis. Contrary to the results for neat PLA, the PLA/TiO_2_/2000 h showed not only the highest degree of crystallinity, but also the worst mechanical properties. There is a critical molecular weight that will maintain the tensile properties of PLA, independent of the degree of crystallinity.

## 4. Conclusions

The purpose of this work was to characterize neat PLA and PLA loaded with three percent by weight of rutile–TiO_2_ after accelerated weathering (UV and moisture conditions) over periods of 500, 1000 and 2000 h. Accelerated weathering exposure had a significant effect on the surface mechanical properties of the PLA samples as a result of degradation. The PLA initially was transparent because of its amorphous character, which changed during the weathering exposure because of the formation of a semi-crystalline structure. The PLA/TiO_2_ nanocomposite exhibited a pale yellow colour after weathering, which was explained through the formation of double C=C bonds from UV degradation. FTIR also showed the generation of a new C=C band for the PLA/TiO_2_ nanocomposite, which proved the photodegradation process of the nanocomposite to be a Norrish II mechanism in the presence of rutile–TiO_2_. The TiO_2_ in the PLA acted as a UV degradation catalyst and irregular cavities and cracks appeared on the sample surface. The roughness and hydrophilic behaviour of TiO_2_ caused better wettability of PLA/TiO_2_ than neat PLA. A hydrophobic surface was also formed in PLA due to the cleavage of ester linkages and reorganization of the polymer chains into a closer crystalline structure, which did not allow water penetration.

PLA’s ability to cold crystallize increased with weathering time and with TiO_2_ nanoparticles mixed into the PLA. With increasing weathering time, increasing numbers of short chains were formed as a result of chain scission, and it was easier for these chains to re-arrange into crystalline fractions. The presence of TiO_2_ nanoparticles not only improved the cold crystallization behaviour of the PLA, acting as nucleating agents, but they also catalyzed the UV-initiated degradation of the PLA, as was also observed in the tensile properties, where a general increase was observed with increasing accelerated weathering time up to 1000 h. However, after 2000 h of weathering exposure, the PLA and PLA/TiO_2_ became extremely brittle and lost their ductile properties. This was the result of the initial increase in and eventual destruction of the crystalline phase in PLA, which was more evident in the PLA/TiO_2_ sample.

## Figures and Tables

**Table 1:** Mechanical properties of PLA and PLA/TiO_2_ nanocomposite collected from AFM analyses.

	Stiffness (mN m^−1^)		Young’s Modulus (MPa)	
	Mean	Width	Mean	Width
**PLA/0 h**	29.7	3.7	29.5	3.6
**PLA/500 h**	70.5	4.6	34.6	2.3
**PLA/1000 h**	79.0	9.6	38.8	4.7
**PLA/2000 h**	63.9	10.4	62.9	10.3
**PLA/TiO_2_/0 h**	59.0	7.2	58.0	7.1
**PLA/TiO_2_/500 h**	65.3	7.9	64.3	7.7
**PLA/TiO_2_/1000 h**	62.0	14.0	61.1	13.7
**PLA/TiO_2_/2000 h**	48.5	6.6	47.8	6.5

**Table 2:** Changes in *M*_n_, *M*_w_ and *Đ_M_* of the PLA and PLA/TiO_2_ samples as a function of accelerated weathering time.

	*M*_n_ (g mol^−1^)	% *M*_n_ Degradation	*M*_w_ (g mol^−1^)	% *M*_w_ Degradation	*Đ_M_*
PLA/0 h	71,500	–	89,500	–	1.3
PLA/1000 h	10,500	85	14,000	84	1.3
PLA/2000 h	6500	91	9000	90	1.3
PLA/TiO_2_/0 h	20,000	–	44,000	–	2.2
PLA/TiO_2_/1000 h	6000	71	20,000	55	3.4
PLA/TiO_2_/2000 h	5000	75	15,000	66	2.9

**Table 3:** DSC data obtained for neat PLA and PLA/TiO_2_ nanocomposite after accelerated weathering degradation.

	Glass Transition	Melting			Cold Crystallization		Degree of Crystallinity
		Enthalpy	Temperature		Enthalpy	Temperature	
Sample	*T*_g_ (°C)	|Δ*H*_m_| (J g^−1^)	*T*_m1_ (°C)	*T*_m2_ (°C)	|Δ*H*_CC_| (J g^−1^)	*T*_CC_ (°C)	*X*_c_ (%)
PLA/0 h	56.9	2.3	147.7		2.2	121.2	0.1
PLA/500 h	59.9	21.6		152.0	19.1	119.8	2.7
PLA/1000 h	60.8	30.4		151.9	27.4	98.9/116.7	3.2
PLA/2000 h	56.7	34.8	147.3	152.7	24.6	107.0	10.9
							
PLA/TiO_2_/0 h	59.8	30.8	146.9	153.1	32.7	109.2	0 *
PLA/TiO_2_/500 h	57.8	33.1	143.8	152.3	28.5	96.4	5.1
PLA/TiO_2_/1000 h	60.4	31.4	144.5	152.5	25.8	94.6	6.2
PLA/TiO_2_/2000 h	56.9	1.8	149.3	153.7	2.4	123.9	0*

* Since degree of crystallinity cannot be negative, the small negative values (within experimental error) were changed to zero.

**Table 4:** Characteristic degradation temperatures for PLA and the PLA/TiO_2_ samples obtained from the TGA and dTGA curves in Figure 13 and Figure 14.

Sample	*T*_5%_ (°C)	*T*_50%_ (°C)	*T*_max_ (°C)
PLA/0 h	332	359	362
PLA/500 h	333	366	370
PLA/1000 h	324	360	365
PLA/2000 h	317	356	364
			
PLA/TiO_2_/0 h	239	307	318
PLA/TiO_2_/500 h	256	306	310
PLA/TiO_2_/1000 h	290	331	334
PLA/TiO_2_/2000 h	295	342	357

**Table 5:** Mechanical properties of neat PLA and the PLA/TiO_2_ after different periods of accelerated weathering.

Sample	σ_break_ (MPa)	ε (%)	E (MPa)
PLA/0 h	25.5 ± 1.2	1.8 ± 0.4	3036 ± 101
PLA/500 h	23.7 ± 2.7	1.7 ± 0.7	2821 ± 301
PLA/1000 h	52.7 ± 1.5	4.0 ± 1.1	1293 ± 975
PLA/2000 h	12.3 ± 4.8	1.3 ± 1.1	1748 ± 431
			
PLA/TiO_2_/0 h	15.3 ± 1.7	0.8 ± 0.3	2646 ± 166
PLA/TiO_2_/500 h	20.9 ± 5.8	1.7 ± 0.6	1861 ± 116
PLA/TiO_2_/1000 h	54.9 ± 3.4	2.4 ± 0.2	2814 ± 321
PLA/TiO_2_/2000 h	5.1 ± 0.0	1.2 ± 0.2	386 ± 97
